# Compromised Item Detection for Computerized Adaptive Testing

**DOI:** 10.3389/fpsyg.2019.00829

**Published:** 2019-04-17

**Authors:** Cheng Liu, Kyung T. Han, Jun Li

**Affiliations:** ^1^Department of Applied and Computational Mathematics and Statistics, University of Notre Dame, Notre Dame, IN, United States; ^2^Center for Research Computing, University of Notre Dame, Notre Dame, IN, United States; ^3^Department of Psychology, University of Notre Dame, Notre Dame, IN, United States; ^4^Graduate Management Admission Council, Reston, VA, United States

**Keywords:** computerized adaptive testing, CAT, compromised item detection, generalized linear model, test security

## Abstract

Item leakage has been a serious issue in continuous, computer-based testing, especially computerized adaptive testing (CAT), as compromised items jeopardize the fairness and validity of the test. Strategies to detect and address the problem of compromised items have been proposed and investigated, but many solutions are computationally intensive and thus difficult to apply in real-time monitoring. Recently, researchers have proposed several sequential methods aimed at fast detection of compromised items, but applications of these methods have not considered various scenarios of item leakage. In this paper, we introduce a model with a leakage parameter to better characterize the item leaking process and develop a more generalized detection method on its basis. The new model achieves a high level of detection accuracy while maintaining the type-I error at the nominal level, for both fast and slow leakage scenarios. The proposed model also estimates the time point at which an item becomes compromised, thus providing additional useful information for testing practitioners.

## 1. Introduction

Due to advances in information technology, continuous testing has been offered for many large-scale testing programs, and test takers can take such exams nearly any time during the year. Although continuous testing provides test takers with considerable flexibility and convenience, it also raises serious security concerns. Individuals who take the test earlier in a testing window could share the items orally or online (e.g., via social media platforms), which would benefit subsequent test takers, jeopardizing the validity and fairness of the test. Studies have shown the severe and negative impact of compromised items (Chang and Zhang, 2002, 2003; Davey and Nering, 2002; McLeod et al., 2003; Yi et al., 2008; Guo et al., 2009; Zhang et al., 2012). Items administered frequently are vulnerable to leakage, and many methods have been proposed to control item exposure to protect test security (Sympson and Hetter, 1985; Stocking, 1994; Stocking and Lewis, 1995; Mills and Stocking, 1996; Hetter and Sympson, 1997; Way, 1998; Chang and Zhang, 2002, 2003; Davey and Nering, 2002; Chen et al., 2003). Sympson-Hetter (SH) method (Sympson and Hetter, 1985) is one of the widely used applications of this strategy. SH method needs an upper cutoff proportion (e.g., 20%) as a parameter. Only those items that are exposed to <20% of all test takers can be selected. This way, SH method is able to prevent the over exposure of an item to the public, which in return reduces the potential damage caused by the item compromise. Since then, researchers have developed many exposure-control strategies following the same direction. Although these methods are generally effective in keeping items from being over exposed, they are only preventive measures and do not directly address the problem of items that have been compromised.

Alternatively, many methods have been developed to proactively detect item preknowledge (McLeod et al., 2003; Belov et al., 2007; Belov and Armstrong, 2010, 2011; Obregon, 2013; Belov, 2014). These methods allow testing practitioners to determine whether an examinee has preknowledge of a set of suspicious items by comparing the estimates of the examinee's ability with and without suspicious items. As examples, Drasgow et al. and Armstrong et al. proposed detection methods using likelihood-based person fit statistics (Drasgow et al., 1985; Armstrong et al., 2007), Levine and Dragsgow proposed another method based on Neyman-Pearson lemma (Levine and Drasgow, 1988), and Belov et al. proposed to use Kullback-Leibler divergence for detection (Belov and Armstrong, 2011). However, in practice, there are two major limitations with the application of these methods. First, it is difficult to identify a set of suspicious items without context or prior information, especially when considering that the item set varies from examinee to examinee. Second, these methods rely heavily on the estimation of the test-taker's ability. When the true ability of the test taker is known, the method usually performs well. In practice, however, the test-taker's true ability is unknown and needs to be estimated. In the case of severe item leakage, the estimation of an individual's ability can become biased, which in turn can lead to inefficiency in detecting item preknowledge.

The above-mentioned proactive methods focus on individual-level test statistics, but in recent years, several item-level sequential methods have been proposed to detect compromised items in computerized adaptive testing (CAT) (Zhang, 2014; Zhang and Li, 2016; Choe et al., 2018). These methods focus on monitoring the change of the expected probability of getting a correct response for an item. To enhance the sensitivity of the detection procedure, they suggest imposing a moving window to select a group of responses from the *n*^*th*^ response to the (*n* + *m*)^*th*^, where *n* is the starting point of the window and *m* is the size of window. Then a hypothesis test is performed to tell whether the expected probabilities of getting an item correct are the same before and within this window. The item will be flagged as compromised if the change is significant. One advantage of this sequential algorithm is that it is computationally fast and hence can be used for real-time detection. Existing studies, however, are limited in several ways and warrant further study. First, these methods require specification of the best window size, which may be challenging for test professionals. Second, the simulation considers only the scenario where the expected probability of a correct response has a sharp increase after an item is compromised. The utility of these methods in the face of a gradual change of the expected probability is unknown. Third, the current sequential detection method can only tell when the leakage is detected but cannot estimate when the item is compromised, from which test practitioners can review the impact of the leakage and re-evaluate test-takers' ability estimation. For example, an item compromised at day *t*_1_ can be detected as an compromised item at day *t*_2_. There is a *t*_2_ − *t*_1_ lag in between before a significant conclusion could be drawn. In this case, *t*_2_ is the detection day, which is known. And *t*_1_ is the compromised day which is not known.

Therefore, there is need for a new, flexible method to account for various item-leaking processes in real life, where compromised items can spread at different rates and item leakage can result from many causes. The new method should be able to detect leakage under different scenarios, and provide an estimate of when an item is leaked.

First, compromised items may spread at different speeds, and the expected probability of correctly response to an item may not jump abruptly to a fixed, high value. For example, a posting on a popular social media website could quickly spread preknowledge of an item, whereas sharing within a small group of acquaintances might result in slower spreading. Therefore, to make the sequential detection approach more robust, it is important to develop a flexible method that takes these underlying dynamics into consideration.

Second, there are many probable causes of item leakage. A common scenario as detailed above could involve a test taker who posts the items received on a website, where future test takers could gain preknowledge on those items. A more severe case is organized item theft, which has been discussed in Yi et al. (2008). In this case, profit-driven organizations may send thieves to take the exam at the early stage in a testing window. The thieves will intentionally memorize the items they receive, aiming to profit from disclosing the items to future test takers. In the “random item leakage" scenario, the time when an item becomes compromised is random. In the “organized item theft" scenario, on the other hand, the leakage usually happens at the very beginning of a testing window. When investigating the performance of detection methods, these different scenarios should be considered.

We therefore propose a new method for proactive detection of compromised items that largely addresses the stated limitations of existing approaches. Our method uses generalized linear modeling with complementary log-log transformation (cloglog) as the link function, and it takes the potential leaking mechanism into consideration. Compared with existing methods, it has the following advantages: (1) It can handle more complicated item leakage mechanisms, both fast and slow; (2) Unlike existing sequential approaches, it does not need a moving window to boost the detection sensitivity, and thus saves the trouble of determining the best window size; instead, it improves the detection accuracy by utilizing complete testing information. (3) It enables the estimation of the “compromise time,” i.e., the time point at which the item was compromised. (4) It is computationally more efficient compared with those item preknowledge detection methods since it does not depend on the selection of suspicious items.

The model is validated by both simulation data and real data in practice. For simulation, the test is performed with simulations under different scenarios and parameters. The simulated datasets are generated as diverse as possible. First, the model we use for simulating data is purposefully designed to differ from our model for leakage detection, in order to test the robustness of our leakage detection method when the underlying leaking mechanism is unknown. Second, our simulation covers two distinct leakage scenarios, organized item theft and random item leakage. Third, for each simulation scenario, we investigated the values of the leakage rate in a wide range, in order to mimic different spread speeds in practice. In addition of all simulation studies above, we also showed an application of our proposed method to a real large-scale testing dataset. Both studies, i.e., simulation and real data, perform well. In our study, an application based on the estimation of the compromised day, *t*_1_, is also proposed, which successfully links the compromised item detection with the person-level preknowledge detection. Simulation results show that *t*_1_ can provide important information for the preknowledge detection in CAT and significantly improve the accuracy of the person's ability estimation.

## 2. Methods

We detect compromised items by monitoring the responses of test takers. When an item is compromised, the expected probability for test takers to answer it correctly will increase. Instead of assuming all responses to always be correct (Yi et al., 2008) or to be a constant probability (Zhang, 2014) immediately after an item becomes compromised in simulation, we propose a gradual change model as a function of time, hereafter referred to as the *leakage model*. The leakage model acknowledges the fact that responses to a compromised item may not always be correct right after its compromise. Instead, as more people are exposed to this compromised item over time, the probability for the item to be correctly answered will gradually increase to 1. This increase can be slow or fast, depending on the rate parameter. When this rate is large, our model will degrade to the previous models mentioned above.

### 2.1. Generalized Linear Model for Detection

In computerized adaptive testing, the probability for a test taker to give a correct response to an uncompromised item can be modeled by a three-parameter logistic (3PL) item response theory (IRT) model (Lord, 1980):

(1) $$\begin{array}{l} P\left(U=1|\theta \right)=c+\left(1-c\right)\frac{1}{1+e^{-1.7a\left(\theta -b\right)}}, \end{array}$$

where θ is the latent ability of a test taker, *a* is the discrimination parameter, *b* is the difficulty parameter, and *c* is the pseudo-guessing parameter. It has been shown (Birnbaum, 1968) that the item will be assigned to test takers whose provisional ability estimate is close to

(2) $$\begin{array}{l} \theta_{0}=b+\frac{\ln\left(1+\frac{\sqrt{1+8c}}{2}\right)}{1.7a}, \end{array}$$

when the maximum item information method is used to select the next item. The expected probability for test takers to answer the item correctly is (1+$\sqrt{1+8c}$)/4. Thus, the probability to answer the target item correctly should fluctuate roughly around this expected probability. When an item is compromised, the expected probability will increase accordingly. In practice, since the ability estimate may not be sufficiently accurate at the beginning of the test, the expected probability to correctly answer the item might not be exactly (1+$\sqrt{1+8c}$)/4 initially. On the other hand, to control the potential damage from item thieves during high stakes exams, an item exposure control component will be implemented, which is a random factor on top of the item selection criterion. Therefore, it is rare that the very item expected to exhibit the largest Fisher information would actually be selected and administered. One of the items with higher information will, though. As the test progresses, however, and if the item pool is sufficiently large, the expected probability should hold, a property that could be used to detect the compromised item. A similar idea was also discussed in Zhang (2014).

In this study, the proposed detection algorithm concerns only the time series of responses of a single item, and all items are treated independently. Unless stated otherwise, we will use a representative item to hereafter illustrate the detection model.

Suppose the expected probability for a test taker to answer this item correctly is 1−π_*t*_ on day *t* (i.e., the probability to get an incorrect answer is π_*t*_). Therefore, the number of incorrect answers *y*_*t*_ should approximately follow a binomial distribution, *y*_*t*_~ Bin (*n*_*t*_, π_*t*_), where *n*_*t*_ is the total number of examinees taking this item on day *t*. Thus, the overall log-likelihood for all *T* testing days for the item of interest is

(3) $$\begin{array}{l} l=\log L=\sum_{t=1}^{T}\left[y_{t}\log\pi_{t}+(n_{t}-y_{t})\log(1-\pi_{t})\right], \end{array}$$

where *t* = 1, 2, ⋯ , *T*. Please note that although we are using days as the unit of *t* for illustration, *t* actually can be any time units. For example, *t* can be hours instead as long as there are enough samples within the time interval.

**Figure 1:**
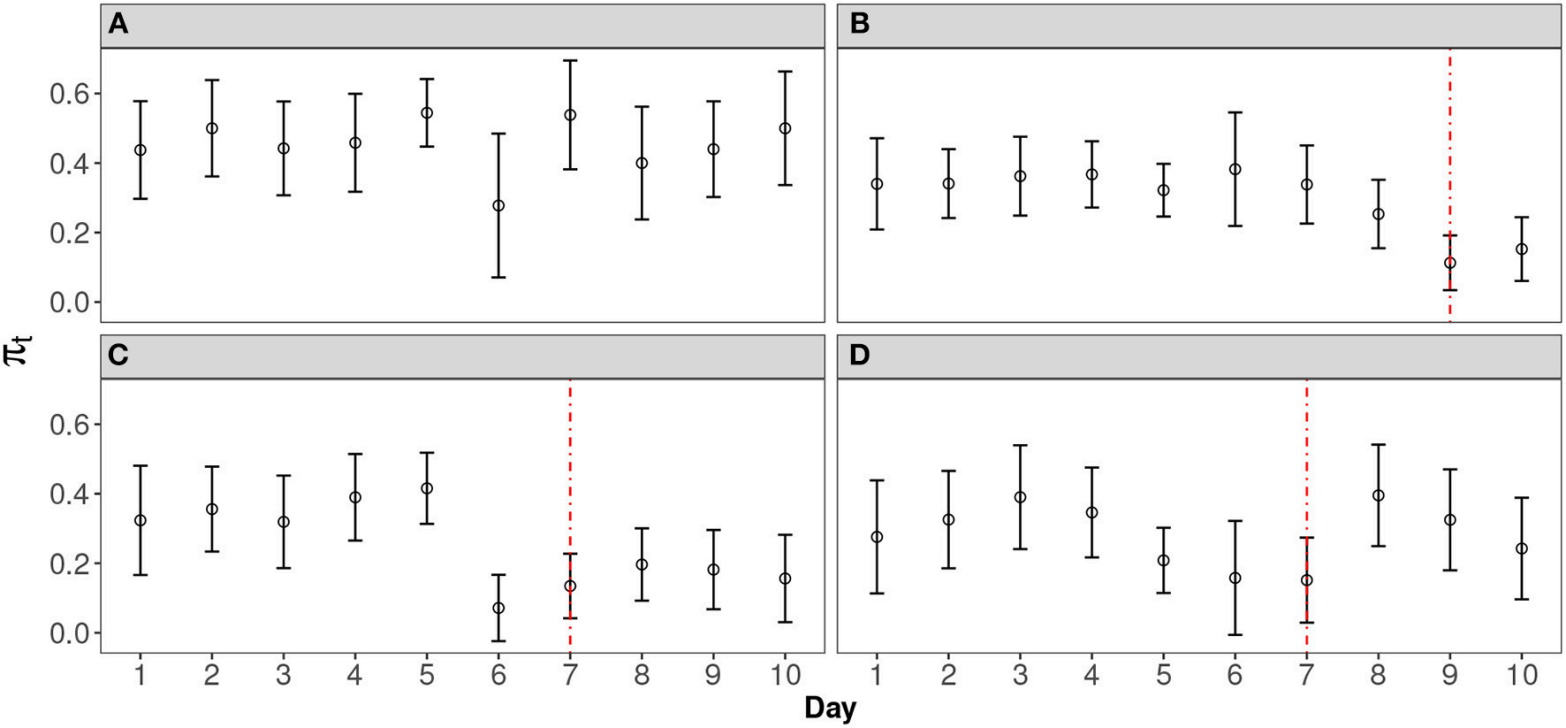
Representative Curves for Different Scenarios. **(A)** Item without any leakage; **(B)** Item with slow leakage; **(C)** Item with fast leakage; **(D)** Item with leakage that goes back thereafter.

In order to design an effective model to detect the leakage pattern in real data, we worked with the researchers in the large-scale testing company in this study. Figure 1 shows four typical curves from the empirical data analysis. These curves are selected from a large-scale operational CAT program that has 2905 items records. The item pool was rotated every 10 days in order to secure the test from item compromise (For more information of this dataset, please see section REAL DATA APPLICATION). The error bars are the 95% confidence interval of the probability of incorrect response of that day, which is calculated by $1.96\sqrt{\frac{\hat{\pi }\left(1-\hat{\pi }\right)}{n}}$ (Agresti, 2013). Figure 1A is an item without leakage. Figures 1B,C represent the leakage with two different leakage rates: slow and fast. Figure 1D shows a scenario where the expected probability goes back up after a significant decrease. For both Figures 1B,C, a sigmoid-shaped pattern curve could be used to model the probability change. For scenario d, although the probability goes back after a significant dip, this scenarios should also be flagged out as well, since: (1) in a continuous test, we can only make our decision based on the data we have at hand. (2) a significant decrease of the expected probability should always be alarmed and carefully investigated, to enhance the security of the test. In this case, a sigmoid-shaped pattern curve can also be used to model the part before it goes back.

**Figure 2:**
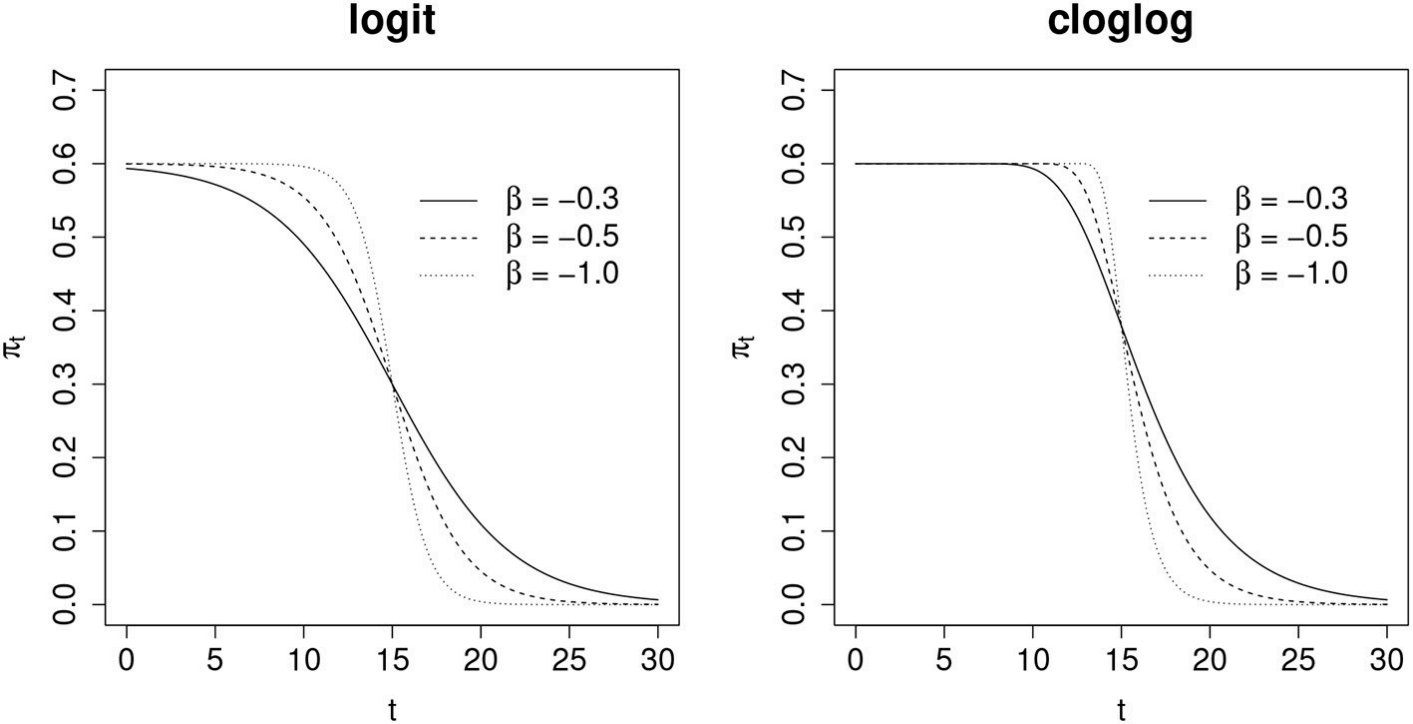
Comparison of link functions with logit and cloglog transformations.

To detect the gradual change of the expected probability, two possible methods could be used to model the probability π_*t*_ as a function of time: logit

(4) $$\begin{array}{l} \text{logit}\frac{\pi_{t}}{\pi_{0}}=\beta \left(t-t_{0}\right), \end{array}$$

or cloglog

(5) $$\begin{array}{l} \text{cloglog}\frac{\pi_{t}}{\pi_{0}}=\beta \left(t-t_{0}\right), \end{array}$$

where π_0_ is the expected probability before leakage and β is a coefficient that controls the speed of the leakage. Here *t*_0_ is the point at which the item is compromised. Figure 2 illustrates the shape of π_*t*_ under different combinations of π_0_ and β for both logit and cloglog functions. In general, π_*t*_ decreases in a sigmoid manner when β is negative, and a larger absolute value of β corresponds to a faster decrease, suggesting a faster leakage of the compromised item. In the beginning, π_*t*_ presumably changes relatively faster than later in the test cycle. This is when some test takers who are eager to obtain preknowledge of the compromised item would like to take the test, since the compromised item likely is still available. In such case, it will induce a faster drop of probability of incorrect response when leakage starts. For this reason, the asymmetry of the cloglog function is favored in this study and will be selected to model π_*t*_. When *t* = *t*_0_, $\pi_{t}=\pi_{0}\left(1-e^{-1}\right)$, which is around 0.63 of the expected probability before leakage. Note that Equation 5 is actually equivalent to the following model

(6) $$\begin{array}{l} \text{cloglog}\frac{\pi_{t}}{\pi_{0}}=\beta t+\alpha . \end{array}$$

That is, $\text{log}\left(-\text{log}\left(1-\frac{\pi_{t}}{\pi_{0}}\right)\right)=\beta t+\alpha$, which gives

(7) $$\begin{array}{l} \pi_{t}=\pi_{0}\left(1-e^{-e^{\beta t+\alpha }}\right). \end{array}$$

For a compromised item, a negative β is expected. Therefore, the problem of detecting a compromised item is converted to performing the following hypothesis test:

(8) $$\begin{array}{l} H_{0}:\beta =0\;vs.\;H_{a}:\beta <0. \end{array}$$

Note that our test is one-sided, since a positive beta corresponds to an increasing π_*t*_, which is not a desired pattern we want to flag out.

In order to perform the hypothesis test, we need $\hat{\beta }$, as well as the estimate of its variance or standard error. $\hat{\beta }$ and ${\hat{\sigma }}_{\beta }$ are obtained via maximum likelihood estimation. Since there is no closed form analytical solution, we use the coordinate-wise Newton-Raphson method to obtain a numerical solution. Compared with the conventional Newton-Raphson that updates all model parameters at the same time, the proposed method successfully avoids the calculation of the inverse of the Hessian matrix, which can be near-singular and cause numerical instability. Our approach has proved to be efficient and stable in all our simulation studies.

Let **Ψ** be the coefficients in our model that need to be estimated. We have $\Psi ={\left(\psi_{1},\psi_{2},\psi_{3}\right)}^{T}$, where ψ_1_ = π_0_, ψ_2_ = β and ψ_3_ = α. In this way, we can use one general symbol **Ψ** to represent all three parameters. The steps of the coordinate-wise Newton-Raphson algorithm are as follows:

1. Initialize model parameters with random starting values. We use $\pi_{0}^{\left(0\right)}=0.5$, β^(0)^ = 0 and α^(0)^ = 0 for all our simulation studies.
2. Update **Ψ** by updating each of its elements consecutively. That is,
  - First, keep $\hat{\beta }$ and $\hat{\alpha }$ unchanged, update ${\hat{\pi }}_{0}$;
  - Then, keep ${\hat{\pi }}_{0}$ and $\hat{\alpha }$ unchanged, update $\hat{\beta }$; and
  - Third, keep ${\hat{\pi }}_{0}$ and $\hat{\beta }$ unchanged, update $\hat{\alpha }$.
  Each of the above updates is given by
  (9) $$\begin{array}{l} {\hat{\psi }}_{k}\leftarrow {\hat{\psi }}_{k}-\frac{\frac{\partial l\left(\text{Ψ}\right)}{\partial \psi_{k}}{|}_{\text{Ψ}=\hat{\text{Ψ}}}}{\frac{\partial^{2}l\left(\text{Ψ}\right)}{\partial \psi_{k}^{2}}{|}_{\text{Ψ}=\hat{\text{Ψ}}}}. \end{array}$$
  (See the Appendix for more details about this equation.)
3. Repeat Step 2 until convergence. The convergence is checked by calculating the change of the log-likelihood after each iteration. If the change is less than a threshold, e.g., 0.001, the model has converged. Then the element of the Fisher information matrix (see Appendix for detail) is
  (10) $$\begin{array}{l} {}[I_{k_{1}}{\;}_{k_{2}}(\pi_{0},\beta ,\alpha )]=-E\left[\frac{\partial^{2}l(\pi_{0},\beta ,\alpha )}{\partial \psi_{k_{1}}\partial \psi_{k_{2}}}\right], \end{array}$$
  where *k*_1_, *k*_2_ = 1, 2, 3. According to the co-factor method of getting the inverse matrix of ***I***, we have
  (11) $$\begin{array}{l} {\hat{\sigma }}_{\beta }=\sqrt{\frac{I_{1}{\;}_{1}\cdot I_{3}{\;}_{3}-I_{1}{\;}_{3}\cdot I_{3}{\;}_{1}}{det(I)}}. \end{array}$$
  Given $\hat{\beta }$ and ${\hat{\sigma }}_{\beta }$, the Wald statistic is given by
  (12) $$\begin{array}{l} z=\frac{\hat{\beta }-\beta_{0}}{{\hat{\sigma }}_{\beta }}\sim N\left(0,1\right). \end{array}$$

When the null hypothesis is rejected (one-sided test), the item will be flagged as compromised. The time from when the item starts to leak, i.e., the “compromised time,” to when a leaked item is flagged, is defined as “detection lag.” This definition of detection lag is the same as that in Zhang (2014) and Shao et al. (2015). Note that the compromised time, denoted as *t*_*c*_, is unknown in real applications. We propose an estimate of it, ${\hat{t}}_{c}$, as the time when π_*t*_ drops to a certain percentage, say ϵ, of π_0_. Based on our model in Equation 7, it is easy to show that

(13) $$\begin{array}{l} {\hat{t}}_{c}=\frac{\text{ln}\left(\text{ln}\frac{1}{1-\epsilon }\right)-\hat{\alpha }}{\hat{\beta }}. \end{array}$$

Especially, we use ϵ = 90%. The bias of this estimate is defined as the “estimation lag”.

Further, the variance of ${\hat{t}}_{c}$ is given by

(14) $$\begin{array}{l} \text{var}({\hat{t}}_{c})=\left(\frac{\partial {\hat{t}}_{c}}{\partial \hat{\alpha }},\frac{\partial {\hat{t}}_{c}}{\partial \hat{\beta }}\right)\cdot \hat{\Sigma }\cdot \left(\begin{array}{c} \frac{\partial {\hat{t}}_{c}}{\partial \hat{\alpha }}\\ \frac{\partial {\hat{t}}_{c}}{\partial \hat{\beta }} \end{array}\right), \end{array}$$

where $\hat{\Sigma }$ is the variance-covariance matrix of ($\hat{\alpha }$, $\hat{\beta }$), and

$$\begin{array}{l} \frac{\partial {\hat{t}}_{c}}{\partial \hat{\alpha }}=-\frac{1}{\hat{\beta }},\\ \frac{\partial {\hat{t}}_{c}}{\partial \hat{\beta }}=\frac{\alpha -\text{ln}\left(\text{ln}\frac{1}{1-\epsilon }\right)}{{\hat{\beta }}^{2}}. \end{array}$$

The elements of $\hat{\Sigma }$ can be easily estimated by the inverse matrix of ***I***, similar to how the variance of β is derived in Equation 11.

### 2.2. Leakage Simulation Model

Our primary goal for introducing a different leakage model is to test the effectiveness of the proposed detection method with unknown underlying leakage rates. The leakage simulation model should have these two features: (1) After the item is compromised, the expected probability to get a correct response will increase; (2) The spread rates of the compromised item may differ across items. In this study, the leaking process is simulated using an exponential function as follows,

(15) $$\begin{array}{l} P\left(\text{a test taker already knows the answer}|\lambda ,t_{0}\right)=1-e^{-\lambda \left(t-t_{0}\right)}, \end{array}$$

where λ is the leakage parameter that regulates how fast the item will be exposed to the public, *t*_0_ is the time point at which the item is compromised, and *t*−*t*_0_ is the time interval since the item was first compromised. The probability for any test taker to have item preknowledge is a function of *t*, or *P*(*t*). Therefore, after integration with the 3PL IRT model, the overall probability for a test taker to correctly answer an item can be captured in a mixture model as follows:

(16) $$\begin{array}{l} P(U=1|\theta ,\lambda ,t_{0})=(1-e^{-\lambda (t-t_{0})})+e^{-\lambda (t-t_{0})}\cdot \\ \;\left[c+(1-c)\frac{1}{1+e^{-1.7a(\theta -b)}}\right]. \end{array}$$

If the test taker already knows the answer to the item due to item preknowledge, the response process is described by the first component of Equation 16, which is $1-e^{-\lambda \left(t-t_{0}\right)}$. Otherwise, the process follows the 3PL IRT model with a probability of $e^{-\lambda \left(t-t_{0}\right)}$. Therefore, the total expected probability for a test taker to correctly answer the item is given by Equation 16. Again, the first component of Equation 16 is a function of time and therefore captures the leakage process, where λ controls the speed of the leakage. For example, given a moderate leakage parameter λ and a compromise time point *t*_0_, responses to the compromised item will contain increasingly more 1 s (i.e., correct responses), as time *t* increases. With a large λ, the responses will almost always be 1 after item compromise, as assumed by previous studies (Yi et al., 2008). Thus, the gradual change model is more flexible and general.

Note that, in this study, simulation model is only used to test the detection model, not to detect the leakage. Compared with the detection model, simulation model is more complex with extra parameters including a person's ability θ. Although we can also use the leakage model to fit the curve and run the hypothesis test thereafter, a simultaneous estimation of person's ability will make the fitting less efficient than the detection model. Since we only care about the detection of probability curve's leakage pattern, the proposed detection model is more straightforward and easier to converge.

## 3. Simulation Design

Simulation studies are conducted to investigate the performance of the proposed detection method. The parameters in our simulation were chosen according to previous publications (du Toit, 2003; Yi et al., 2008; Zhang, 2014). A total of 400 randomly generated items serve as the item pool. The underlying IRT model is 3PL with item parameters generated as follows:

(17) $$\begin{array}{l} a\sim \text{lognormal}\left(0,0.5\right),\;b\sim \text{N}\left(0,1\right),\;c\sim \text{U}\left(0,0.25\right). \end{array}$$

The discrimination parameters are generated by lognormal distribution. An exposure control procedure is implemented to prevent items from being over-exposed and to protect test security. The exposure rate for an item is defined as

(18) $$\begin{array}{l} \rho =\frac{\#\text{ of times an item has been administered}}{\text{total number of test takers}}. \end{array}$$

In this study, the exposure control parameter is set to be 0.2, meaning only items with exposure rate lower than 0.2 are eligible for administration. Items in the bank belong to three content areas with percentages 40, 30, and 30%, respectively. Test length is set at 40. A content control procedure is implemented in the simulation to ensure that 40, 30, and 30% of items are selected from each content area for every test taker (i.e., 16, 12, and 12, respectively). The item with the lowest exposure rate in the desired content area will be selected as the first item for the incoming test taker. A sample of 500 test takers (θs) are generated each day to take the exam, whose abilities follow standard normal distribution. The simulation is replicated 10 times and all the distribution figures presented in the remainder of this paper are generated based on results aggregated over replications.

A test item could become compromised for a variety of reasons. The interest of this study is to investigate the effectiveness of the detection algorithm in general. In order to achieve this goal, we studied two common scenarios, which form the core of this paper:

1. Organized item theft. Organized item theft is one of the most severe item leakage scenarios in computer-based testing (Yi et al., 2008). Since organized theft usually occurs early in one testing window for maximal gain, 20 item thieves are randomly generated in the first 4 days of the exam cycle. A simple assumption that each thief can randomly remember 10 items is used here, although professional item thieves could remember more. The items will be treated as compromised when they are remembered. Leakage simulation model will be applied thereafter.
2. Random item leakage. Some test takers simply share the items that they have memorized with the public. In this instance, the leakage could occur any time. For the purpose of this study, 20 such item sharers are randomly selected during one testing window. A testing window is so defined that no item pool maintenance such as rotation or replenishment occurs within that window. In other words, the item bank remains the same throughout the window. In this study, the testing window is set to be 30 days (one month). In practice, this number highly depends on the operation of testing company. It might not be a fixed value even for the same test. For simulation, we use monthly rotation to demonstrate the methodology. On average, we assume each item sharer could remember at random 10 out of the 40 items and share these with the public. Usually the motivation to share items is weak near the end of a testing window. For this reason, this simulation study assumes that such random sharing behavior happens only in the first 25 days.

For each test taker, the first item is selected from the item bank that has the lowest exposure rate at that time from the desired content areas. The probability of the test taker to give a correct answer to the target item is calculated based on the mixture leakage model (Equation 16). Then a uniform distributed random number will be generated within (0, 1). If its value is less than the mixture probability, the response will be 1 (i.e., a correct answer). Otherwise, the response will be 0. The expected a posterior (EAP) method (Bock and Mislevy, 1982) is used to estimate an individual's ability given this person's previous responses. After the first item, the standard CAT procedure using maximum item information method (Lord, 1980) is adopted to select the next item according to the estimated $\hat{\theta }$.

In some extreme cases, the probability of getting an item correctly after it is compromised is 1 for all test takers. But, in practice, item leakage could be a gradual change process. In this study, leakage parameter λ (see Equation 15) are set to be 0.05, 0.1, 0.3, 0.5, 0.7, 1, and 1.5 to regulate the differential speed of item leakage. When λ is large, e.g., λ = 1.5, the simulation represents a severe leakage scenario, in which nearly all responses will be correct once an item has been compromised.

## 4. Results

As illustrated in the Method section, the proposed leakage detection model intentionally uses Equation 7, which differs from the true underlying model (Equation 16) that is used to generate the item responses. Parameter λ controls the speed of leakage. The days to reach the probability's half-drop can be approximately estimated by $-\frac{1}{\lambda }\log\left(0.5\right)$, which are around 14, 1.4, and 0.5 when λ is 0.05, 0.5, 1.5, respectively.

### 4.1. Organized Item Theft

As mentioned earlier, this study assumes that all item thieves have taken the test in the first 4 days within a testing window. Table 1 shows the results of detection accuracy and corresponding type-I error in this case. Detection accuracy is defined as the proportion of compromised items correctly identified as such. Type-I error is the proportion of uncompromised items that are incorrectly identified as compromised items.

(19) $$\begin{array}{l} \text{accuracy}=\frac{\#\text{ of compromised items that are correctly flagged}}{\#\text{ of total compromised items}} \end{array}$$

(20) $$\begin{array}{l} \text{type}-\text{I error}=\\ \frac{\#\text{ of items that are incorrectly flagged as compromised items}}{\#\text{ of total uncompromised items}} \end{array}$$

**Table 1:** Detection accuracy and Type-I error for organized item theft (standard error is given in parenthesis).

**Leakage rate (λ)**	**Accuracy (%)**	**Type-I error (%)**
0.05	93.70 (0.54)	4.49 (0.37)
0.10	99.86 (0.09)	6.56 (0.56)
0.30	99.93 (0.06)	7.67 (0.70)
0.50	99.43 (0.27)	4.09 (0.76)
0.70	99.61 (0.13)	4.89 (0.74)
1.00	99.04 (0.16)	4.99 (0.34)
1.50	98.85 (0.26)	4.32 (0.83)

**Figure 3:**
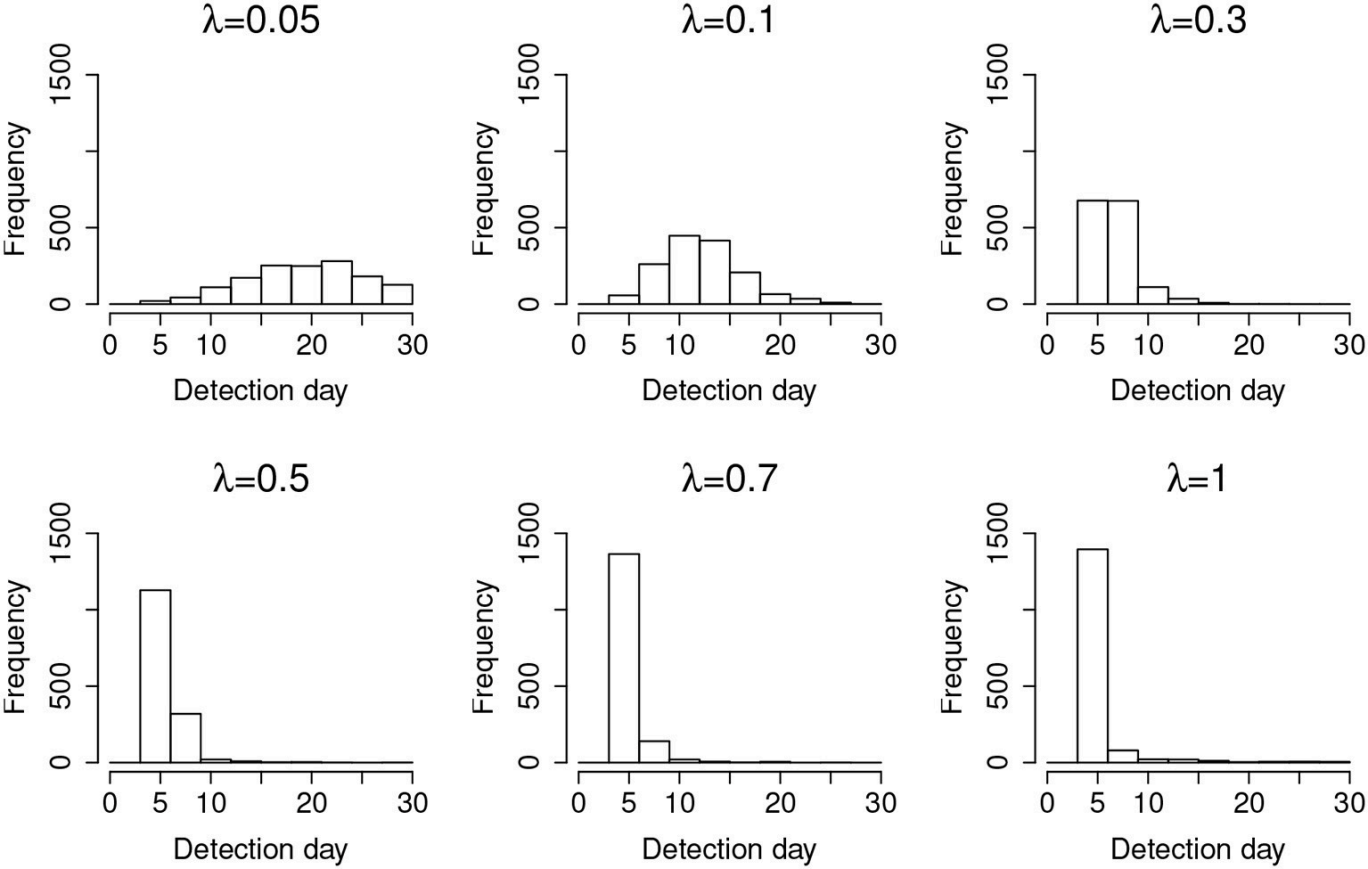
Distribution of the detection day for organized item theft.

For a desired 95% confidence interval, the detection accuracy is about 99% for those λs larger than 0.05. When λ = 0.05, the detection accuracy drops to 93.70%. This is because λ = 0.05 represents a very slow leakage process, which is hard to detect within the 30-day window. On the other hand, the type-I errors for all λs are well controlled at ~5%, consistent with the desired 95% confidence interval. Figure 3 represents the distribution of the detected date of item compromise for different λs within the 30-day window. Overall, when λ is small, the distribution shows large variability. When λ is large, the detection is rather accurate, i.e., pinpointing compromise within the first 4 days. In addition, when λ = 0.05, the distribution of detected dates for compromised items shows a significant portion of items being truncated by the end of the 30-day testing window. Figure 3 provides a direct explanation why the detection accuracy is only 93.70% when λ is small. It is expected that, given more time, more compromised items would be detected and the detection accuracy would be higher.

Table 2 shows the detection lag and the estimation lag. According to Table 2, the mean detection lag is more than 10 days when λ is small (0.05 and 0.10 in our study). When λ ≥ 0.3, the detection lag drops to ~4 days. The probabilities for a coming test taker to have preknowledge of the item are estimated using Equation 15 with λ and average detection lag. Although the detection lag for small λ is large, the impact of the large lag is actually smaller than the cases with large λs. On the other hand, the estimation lag is about 1 day for all λs. All the above results are obtained using ϵ = 90% in Equation 13. When ϵ = 85% or ϵ = 95% is used, the estimation lag is slightly worse yet still quite comparable.

**Table 2:** Detection lag and estimation lag for organized item theft (standard error is given in parenthesis).

**Leakage rate (λ)**	**Detection lag (days)**	**Estimation lag (days)**	**Probability of preknowledge**
0.05	17.47 (0.16)	0.61 (0.19)	0.58
0.10	10.61 (0.11)	–0.46 (0.11)	0.65
0.30	4.69 (0.06)	–0.95 (0.04)	0.76
0.50	3.47 (0.05)	–1.07 (0.03)	0.82
0.70	3.03 (0.05)	–1.13 (0.02)	0.88
1.00	3.11 (0.08)	–1.07 (0.02)	0.96
1.50	4.96 (0.14)	–1.00 (0.01)	1.00

**Figure 4:**
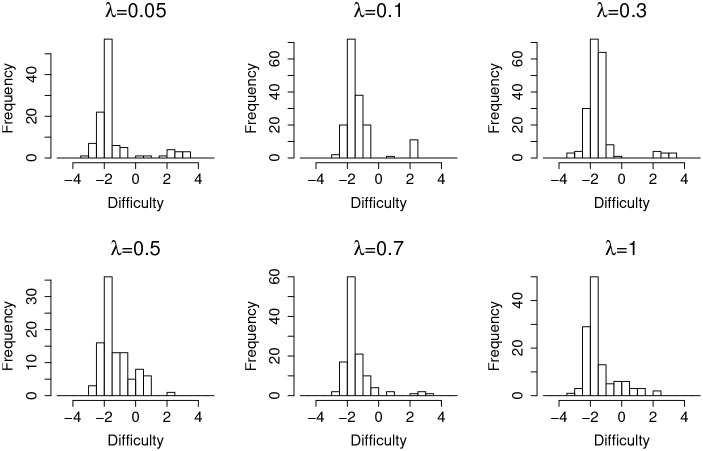
Item distribution of Type-I error items for organized item theft.

Figure 4 shows the distribution of items that are incorrectly flagged as compromised (type-I error) as a function of item difficulty, at different leakage rates. It suggests that, in general, easier items are much more prone to type-I error. Since most of the test takers could correctly answer an easy item without any preknowledge, the majority of the responses will be 1 s regardless of item leakage. In this case, the detection algorithm will capitalize on the randomness of item responses, which in turn triggers more false positives.

**Figure 5:**
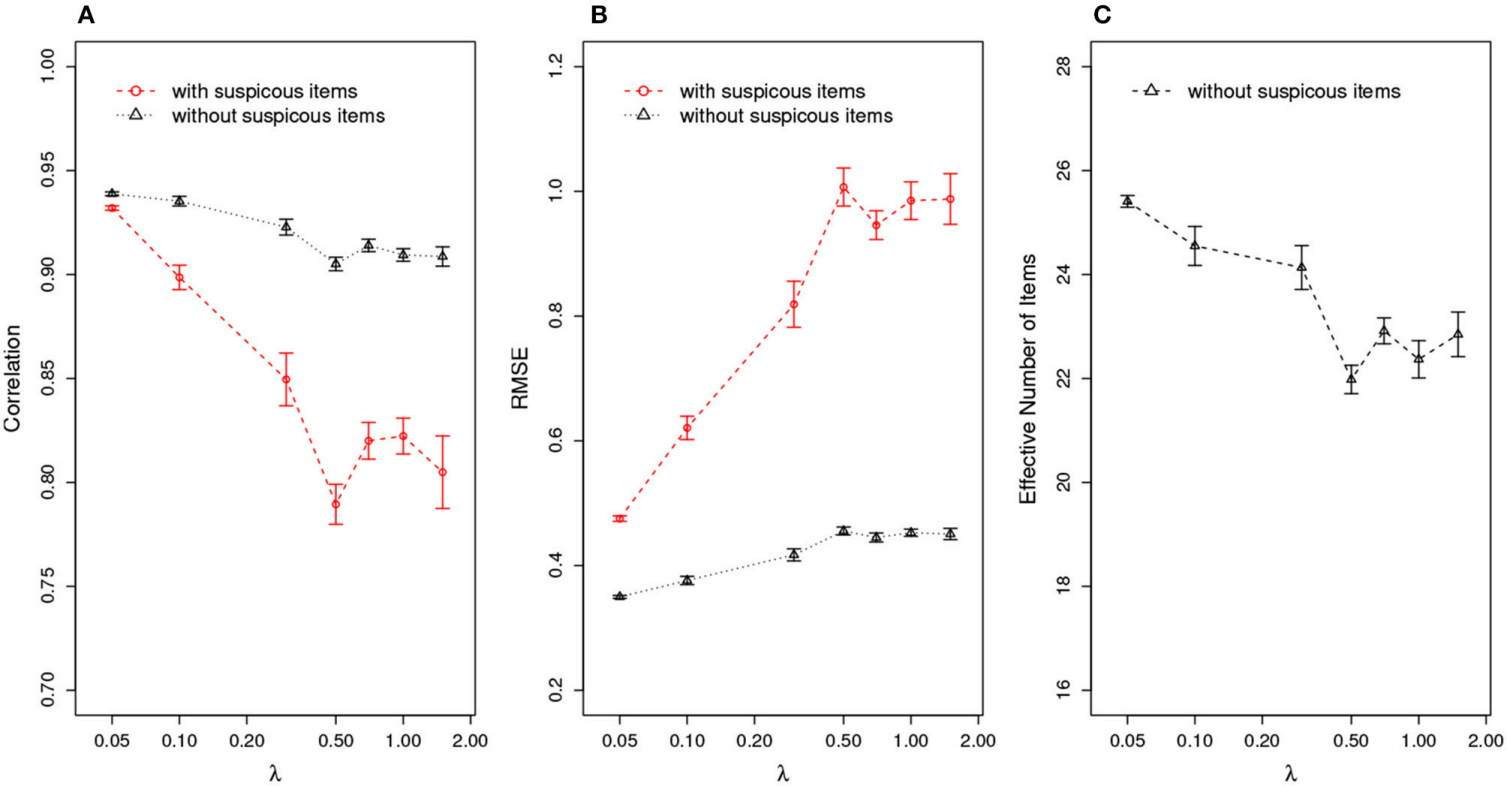
Ability Estimation with/without Suspicious Items for Organized Item Theft. **(A)** correlation of estimated $\hat{\theta }$ with true θ; **(B)** RMSE of estimated $\hat{\theta }$; **(C)** effective number of items after removing suspicious items. (X axis is log scale).

Further, given the estimation of an item compromise point, test practitioners could re-evaluate a test-taker's ability by removing the responses to the suspicious items from ability estimation. Suspect items are defined as those compromised items administered to test takers who take the test after the item compromise point. For example, if an item is flagged as being compromised on day 3 and it was assigned to a test taker on day 4, this item will be classified as a suspicious item for that test taker. Figure 5 compares the ability estimation with and without suspicious items. The results indicate that, after removing the suspicious items, the ability estimation is significantly better than the one in which all items are used, as evidenced by higher correlation between true and estimated ability, and smaller RMSE in ability estimates. Figure 5C shows the effective number of items for ability estimation, meaning the number of items left after removing suspicious items. Under the organized item theft scenario, the effective test length could drop to as low as 22 items, which is about half of the original test length 40. Since the number of effective items will affect the accuracy of the ability estimation, it is expected that the estimation should be more accurate when λ is small (when there are more effective items left, as shown in Figure 5C), corresponding to an increase in correlation and decrease in RMSE as shown in Figures 5A,B.

### 4.2. Random Item Leakage

**Figure 6:**
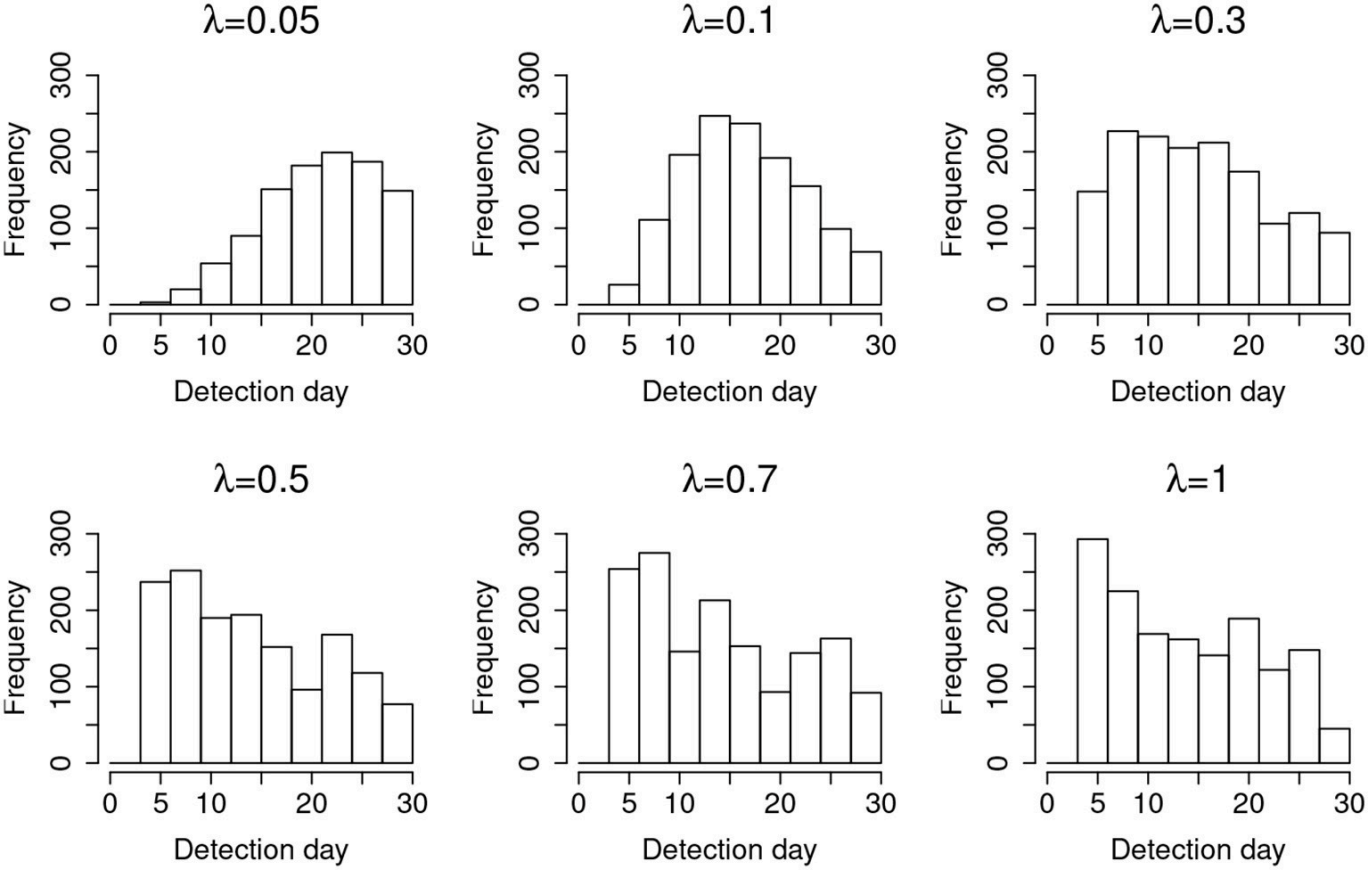
Distribution of the detection day for random item leakage.

Results from studying the random item leakage conditions show common patterns with those of the organized item theft conditions. However, unlike the scenario of organized item theft, random item leakage does not always start at the beginning of the item bank rotation. The leakage can occur any time before the rotation of the item pool. Therefore, more data are available before the leakage. This part of the study examines how the model performs under such a scenario. Table 3 shows the detection power when random item leakage happens in the first 25 days. As with organized item theft, the detection accuracy is very close to 100% when λ ≥ 0.3. Due to the shortage of detection time when λ is small, the detection accuracy drops significantly given the 30-day simulation window. Therefore, it is difficult to effectively detect slow leaking items when the compromise date is close to the end of the test cycle. For example, if a test taker decides to share the test items assigned to him/her at day 25, π_*t*_ will not change much from day 25 to 30 when λ is small. When λ is large, however, a significant change of π_*t*_ could still be observed within 5 days. Figure 6 shows the distribution of the detection days under the random leakage conditions. Compared with Figure 3, the distribution has large variability. The truncation of the detection day is severe in this case when λ is small.

**Table 3:** Detection accuracy and Type-I error for random item leakage (standard error is given in parenthesis).

**Leakage rate (λ)**	**Accuracy (%)**	**Type-I error (%)**
0.05	67.63 (2.61)	2.02 (0.44)
0.10	87.00 (1.98)	4.54 (0.43)
0.30	96.64 (1.03)	5.14 (0.92)
0.50	98.80 (0.33)	4.67 (1.27)
0.70	99.13 (0.50)	4.85 (0.50)
1.00	99.79 (0.10)	5.22 (0.90)
1.50	99.74 (0.14)	4.01 (0.83)

**Figure 7:**
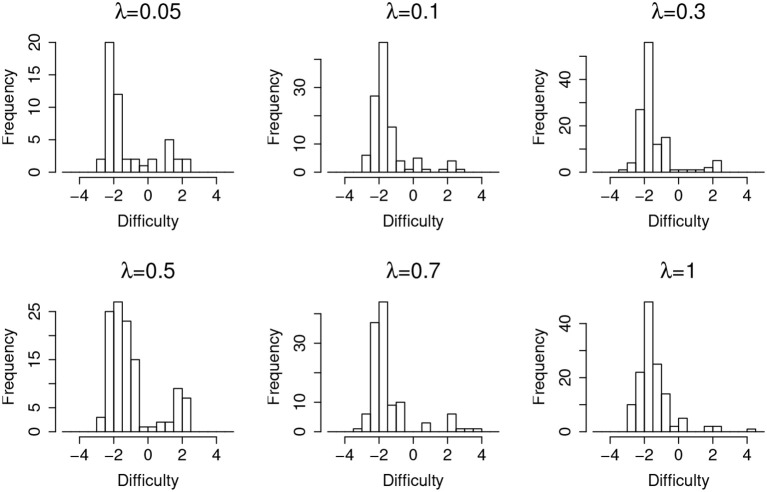
Item distribution of Type-I error items for random item leakage.

Table 4 shows the detection lag and the estimation lag of the compromise time for random item leakage. The detection lags are about 1 day shorter than in the case of organized item theft, which suggests the model works better for the random leakage scenario. This is because, for the organized item theft scenario, the detection could not start until day 4, since all the item theft is assumed to happen in the first 4 days. As a consequence, for those items compromised at day 1, the earliest detection day is day 4 (i.e., the lowest possible lag is 3 days). On the other hand, although the assumption of organized item theft affects the detection lag, it does not significantly affect the estimated compromise time very much. The method used to estimate the item compromise time shows similar results in both scenarios, which is about 1 day. Similar to Figures 4, 7 also shows that most of the type-I errors are related to those easy items under random leakage scenario as well.

**Table 4:** Detection lag and estimation lag for random item leakage (standard error is given in parenthesis).

**Leakage rate (λ)**	**Detection lag (days)**	**Estimation lag (days)**	**Probability of preknowledge**
0.05	12.42 (0.15)	0.66 (0.16)	0.46
0.10	7.76 (0.08)	0.18 (0.08)	0.54
0.30	3.88 (0.05)	–0.90 (0.05)	0.69
0.50	3.00 (0.04)	–1.00 (0.04)	0.78
0.70	2.66 (0.04)	–1.16 (0.04)	0.84
1.00	2.38 (0.04)	–1.14 (0.03)	0.91
1.50	2.64 (0.07)	–1.17 (0.03)	0.98

**Figure 8:**
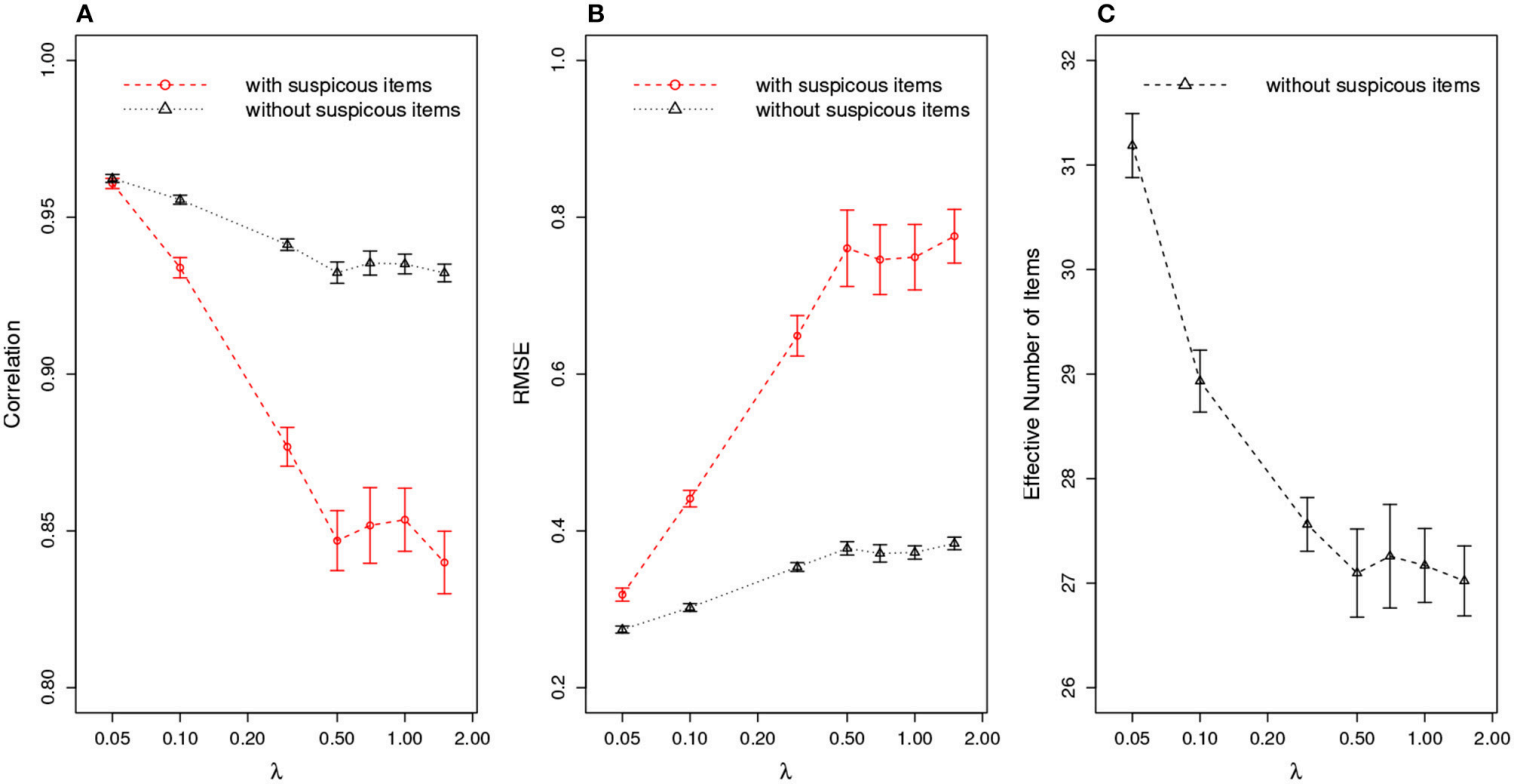
Ability Estimation with/without Suspicious Items for Random Item Leakage. **(A)** correlation of estimated $\hat{\theta }$ with true θ; **(B)** RMSE of estimated $\hat{\theta }$; **(C)** effective number of items after removing suspicious items. (X axis is log scale).

Figure 8 compares the ability estimation with and without those suspicious items. Similar to the scenario of organized item theft, the ability estimation is significantly improved after removing suspicious items. Figure 8C shows that the effective test length is about four items longer than the other scenario above.

## 5. Real Data Application

In this study, we demonstrate the use of the proposed methods with real data from a large-scale operational CAT program that offers continuous testing. Item response data for about 10 days from two operational item pools are used for the analysis. There are 2905 items in total and only 32 items are flagged as being compromised, with nominal alpha level at 0.05. This result indicates that this operational testing program is rather secure, with only slightly over 1% (32 out of 2905) of potential leakage detected. Although the nominal Type-I error is 0.05, the empirical alpha level may be different due to many factors, e.g., the short testing interval (10 days). For all four typical curves illustrated in Figure 1, dashed lines indicate when the leakage is detected by our proposed method. There are 14, 4, and 8 flagged items, respectively, in Types b, c and d. Since the method is designed to monitor the probability change sequentially, information after the detection (dashed line) is not used for fitting the model. In contrast to Types b and c, Type d items are challenging to interpret. They may not necessarily be compromised but the large fluctuation that triggered the flag for these items suggests testing practitioners should investigate further these items closely in case there is a leakage. The Type d scenario might indicate group preknowledge of the item of interest. One conjecture is that those who cheat often also attempt to time the item pool rotation. For example, they try to schedule and take tests as soon as they have certain amount of preknowledge of items after the pool rotation, to improve their chance of seeing some of the leaked items before the pool rotates again. Since future responses are not foreseen and we can only draw conclusions based on the response data currently at hand, in practice, once an item is flagged (no matter if it is Type b, c, or d), it should be removed from the item pool at least temporarily. When an item is flagged, one cannot be sure if its probability curve will eventually go back up or not. Test practitioners need to balance between being conservative and liberal. Given the importance of test security, if only a small number of items are flagged as being potentially compromised, the cost to exclude those items from test administration is limited so the choice is obvious.

## 6. Model Comparison

We compare our proposed detection method with the existing method (Zhang, 2014), using both simulation data and real data. Zhang's sequential model requires setting of two parameters, the length of burn-in period and the size of the moving window. We follow Zhang's simulation study and set them as 150 and 50, respectively. Our proposed cloglog detection model, on the other hand, contains no tuning parameters and the detection starts automatically at day 4 since the model has three coefficients to fit.

First, we apply Zhang's detection model to our simulation data with a leakage process taken into consideration. Tables 5, 6 summarize the results on the random leakage scenario and the organized theft scenario, respectively. Firstly, we notice that in all scenarios, the type-I error is much larger than the α value, the nominal type-I error. For example, under α = 0.05, the type-I error is larger than 70% under every simulation scenario. This means that Zhang's method did a poor job in controlling the type-I error. Secondly, even if we ignore the inflation of type-I error, Zhang's method still has a lower power than our method. The difference in power of the two methods is especially large when the leakage rate is small. For example, under the random leakage scenario with a low leakage rate (λ = 0.05), the power of our method is 67.63%, while Zhang's method is 37.44% when the type-I error is reasonably low (<2% achieved under α = 0.0001). This agrees with our expectation: sliding-window-based methods are not as efficient in capturing slow leakage as methods that describe and utilize the shape of probability change.

**Table 5:** Application of Zhang's sequential method to random leakage scenario.

**Leakage rate**	**α = 0.05**		**α = 0.01**		**α = 0.001**		**α = 0.0001**	
	**Accuracy(%)**	**Type-I(%)**	**Accuracy(%)**	**Type-I(%)**	**Accuracy(%)**	**Type-I(%)**	**Accuracy(%)**	**Type-I(%)**
0.05	99.68	89.87	94.50	48.36	73.95	8.90	37.44	1.58
0.1	99.87	84.29	98.08	42.37	91.78	9.75	71.31	2.95
0.3	99.40	77.97	99.31	39.00	97.18	9.30	90.51	2.98
0.5	99.93	77.89	99.74	37.83	98.01	8.56	92.05	2.75
0.7	99.94	74.96	99.68	32.80	97.78	6.79	93.06	2.46
1.0	100.00	76.40	99.41	35.60	96.81	8.11	91.46	2.46
1.5	99.93	74.40	99.01	32.38	95.74	7.47	91.36	1.98

**Table 6:** Application of Zhang's sequential method to organized theft scenario.

**Leakage rate**	**α = 0.05**		**α = 0.01**		**α = 0.001**		**α = 0.0001**	
	**Accuracy(%)**	**Type-I(%)**	**Accuracy(%)**	**Type-I(%)**	**Accuracy(%)**	**Type-I(%)**	**Accuracy(%)**	**Type-I(%)**
0.05	100.00	81.81	99.81	36.89	91.75	9.04	44.80	2.63
0.1	99.93	72.07	98.86	36.41	95.32	9.66	56.41	3.89
0.3	99.93	75.37	99.93	40.17	92.22	11.56	64.05	4.51
0.5	99.93	76.72	99.63	41.27	90.39	11.92	61.03	3.10
0.7	99.68	79.17	99.65	42.74	83.88	10.96	60.33	2.80
1.0	99.54	76.60	96.63	40.86	80.73	9.96	61.54	2.56
1.5	99.19	77.48	93.23	43.83	74.96	10.34	56.59	1.88

**Figure 9:**
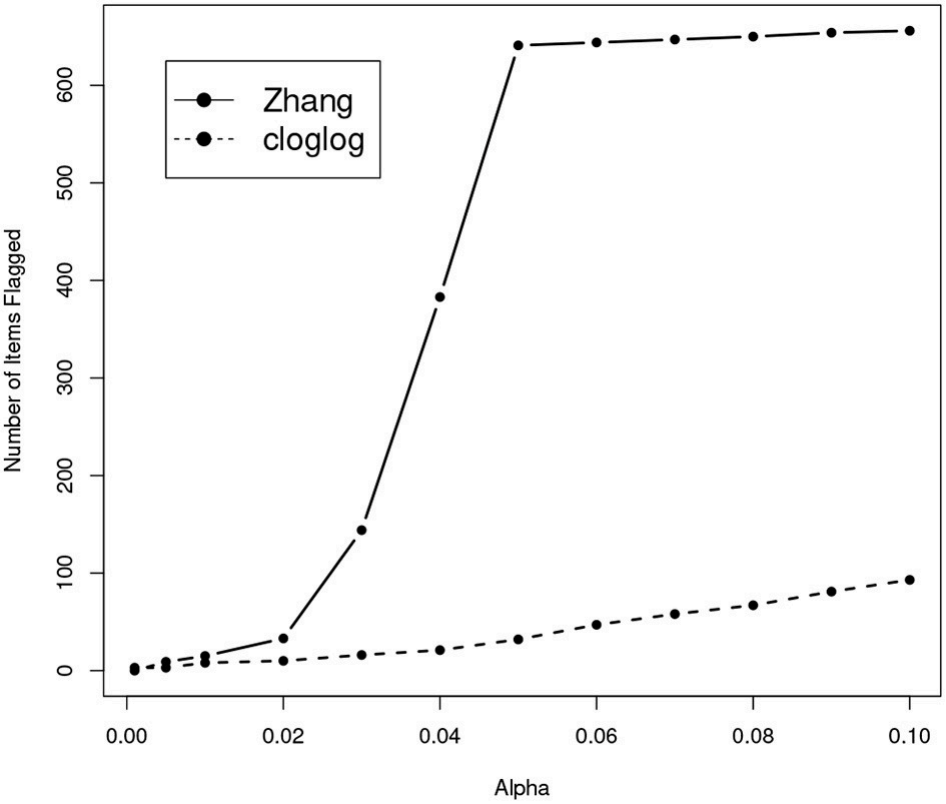
Number of items that are flagged as compromised with different α for two models.

We also apply Zhang's sequencial method to the real dataset we used in the section of “Real Data Application”. Figure 9 shows how the number of items flagged as compromised increases as the nominal type-I error level increases. Strange enough, while the number of leaked items flagged by our method shows a roughly linear increase as the nominal α value increases, the number of leaked items flagged by Zhang's method shows a dramatic increase when the nominal type-I error is in the range of 0.02 and 0.05. When α is 0.05, Zhang's method flagged over 600 items as compromised, which is more than 20% of the entire item pool. Although it is hard to make any conclusive statement on a real dataset with no knowledge about which items are truly leaked, based on results from our simulation study it is not completely unreasonable to suspect that this high rate of detection may be due to severe inflation of the type-I error.

## 7. Conclusion and Discussion

In this study, we have proposed a general detection model that considers the practical dynamics of the item leaking process. The method shows, through all our simulation studies, a strong detection power for various leakage rates with well-controlled type-I error. The model also provides a way to estimate the time point at which an item is compromised, which may be helpful for testing practitioners to better secure the testing process.

The goal of our method is to detect the item leakage for various leakage rates with unknown underlying leaking processes. Therefore, the simulation model of the leakage is purposefully designed to differ from the compromised item detection model. The results show that the proposed model for detection performs very well under such scenarios, which is a strong indicator of the generality and powerfulness of our detection method. Estimates of both detection accuracy and detection lag are close to the expected value when the leakage rate is not too small. When the leakage is very slow, we have observed a longer detection lag time. The impact of this lag, however, can be quite mild in real data applications: When λ is small, the change in probability of getting a correct answer is not large even with a relatively large detection lag. Further, this lag is inevitable: Determining whether an item is compromised when the leakage is slow is intrinsically difficult, no matter what method is used.

The assumption of the detection model is that, given an infinitely long testing window, all test takers eventually will be aware of the compromised item and hence be able to respond correctly, which is implicit in Equation 7. In practice, it may be the case that some portion of test takers will not gain any preknowledge of the items, no matter the length of long the testing window. Therefore, the probability of correctly answering a compromised item ultimately may never reach 100%. In that case, we can generalize our method to cover such a scenario as follows:

(21) $$\begin{array}{l} \text{cloglog}\frac{\pi_{t}-\pi_{e}}{\pi_{0}-\pi_{e}}=\beta t+\alpha \Rightarrow \pi_{t}=\pi_{e}+\left(\pi_{0}-\pi_{e}\right)\left(1-e^{-e^{\beta t+\alpha }}\right), \end{array}$$

where one more parameter π_*e*_ is introduced to represent the expected upper asymptote after the item has been compromised. The π_*e*_ could be any value between [0, π_0_]. When π_*e*_=0, the model reduces to the simplified model in Equation 7. It will be our future work to implement this more general model.

The validation of the model was performed both by simulation and real data. Through the simulation study we were able to generate different leakage dynamics and test the effectiveness of our proposed method in these scenarios. Note that, although both models control the leakage speed, the parameter β in the detection model is not mathematically related to the λ in the simulation model. Actually, our proposed method essentially focuses on detecting the leakage pattern. As long as the overall pattern of the expected probability curve is similar to what we proposed, the method should work. We also applied the method to real data to demonstrate its utility in practice.

The simulation study shows that our proposed method is powerful and reliable when applied to CAT using the maximum item information method for item selection. But the method is not limited to a particular item selection method. Letting *g*(θ) represent the distribution of an individual's ability assigned to an item, the expected probability for a correct response of this item is:

(22) $$\begin{array}{l} E\left(P\left(\theta |a,b,c\right)\right)=\int_{-\infty }^{\infty }P\left(\theta |a,b,c\right)g\left(\theta \right)d\theta =F\left(a,b,c\right). \end{array}$$

The expected probability, therefore, does not depend on the distribution of θ. Different item selection algorithms provide different *g*(θ), but will still lead to a constant expected probability of a given item. Furthermore, this method can also be applied to a non-CAT scenario. Compared with the CAT scenario, where individual's abilities fluctuate around the item difficulty, the distribution of non-CAT is expected to be more spread out. Since, for the CAT scenario, only test takers whose estimated abilities are close to a certain value (see Equation 2) will be assigned to this item, the distribution of their abilities is less variable than the original *g*(θ). Therefore, it is expected that more data are required if this detection model is applied, in order to draw a statistically significant conclusion.

Although the time unit in this study is set at the day level, its selection is very flexible and can be set at finer levels if necessary. The best way to select a time unit depends on the property of the test of interest and expert judgment of experienced testing practitioners. For example, given a large number of scheduled test takers per day, the time unit could be further divided by hourly increments. This would allow for more time points to be used for model fitting, subsequently leading to higher detection sensitivity. On the other hand, instead of aggregating the data by time, one could also choose to aggregate the data by a fixed number of item responses, e.g., every 20 responses. In addition, the type-I error for the hypothesis test is set to be 0.05 in this study, following convention. In practice, the cutoff could be chosen per test practitioners' preference as well.

Our study shows that the ability estimation $\hat{\theta }$ can be significantly improved by removing the responses of suspicious items. A potential future study is to apply our method to determine whether or not an examinee has preknowledge of some test items. This could be accomplished by comparing the ability estimates derived with and without the suspicious items (i.e., items that are flagged by our method). As mentioned in the introduction, many individual-level preknowledge detection methods essentially compare the ability estimates obtained from the secure vs. suspicious test items (Belov, 2016). Our method allows practitioners to identify a set of suspicious items, which is critical to the success of those individual-level detection methods. A retest may be necessary for those test takers whose ability estimates significantly differ with and without suspicious items.

Comparing with the existing sequential method (Zhang, 2014), our method shows large performance boosts in all our simulation data with a variety of leakage rates. On real data, although it is impossible to evaluate and compare the true performance of different methods, our method does not show the apparently erroneous shape of the curve of how the number of flagged items changes according to the nominal Type-I error. Further, Zhang's method asks the user to set the window size parameter, which can be almost unfeasible, our method does not have such tuning parameters.

## Author Contributions

CL and JL developed the method. CL did the simulation study and analysis on real data. KH provided the real data and did the real data analysis with CL and JL. CL, JL, and KH wrote the manuscript.

### Conflict of Interest Statement

The authors declare that the research was conducted in the absence of any commercial or financial relationships that could be construed as a potential conflict of interest.

## Appendix

According to the chain rule of differentiation, the deduction could be divided into two parts: First derive the log-likelihood toward π_*t*_; then calculate the derivative of π_*t*_ toward model parameters π_0_, β and α. Therefore, for convenience, let $\Psi ={\left(\pi_{0},\beta ,\alpha \right)}^{T}$ and let

(A1) $$\begin{array}{l} l=\log L=\sum_{t=1}^{T}\left[y_{t}\log\pi_{t}+(n_{t}-y_{t})\log(1-\pi_{t})\right]=\sum_{t=1}^{T}f(\pi_{t},n_{t},y_{t}), \end{array}$$

where *f*(π_*t*_, *n*_*t*_, *y*_*t*_) is function of π_*t*_, *n*_*t*_, *y*_*t*_. Then we have,

(A2) $$\begin{array}{l} \{\begin{array}{c} \frac{\partial l}{\partial \psi_{k}}=\sum_{t=1}^{T}\frac{\partial f(\pi_{t},n_{t},y_{t})}{\partial \pi_{t}}\frac{\partial \pi_{t}}{\partial \psi_{k}}\\ \frac{\partial^{2}l}{\partial \psi_{k}^{2}}=\sum_{t=1}^{T}\left[\frac{\partial^{2}f(\pi_{t},n_{t},y_{t})}{\partial \pi_{t}^{2}}{\left(\frac{\partial \pi_{t}}{\partial \psi_{k}}\right)}^{2}+\frac{\partial f(\pi_{t},n_{t},y_{t})}{\partial \pi_{t}}\frac{\partial^{2}\pi_{t}}{\partial \psi_{k}^{2}}\right]\\ \frac{\partial^{2}l}{\partial \psi_{k_{1}}\partial \psi_{k_{2}}}=\sum_{t=1}^{T}\left[\frac{\partial^{2}f(\pi_{t},n_{t},y_{t})}{\partial \pi_{t}^{2}}(\frac{\partial \pi_{t}}{\partial \psi_{k_{1}}}\frac{\partial \pi_{t}}{\partial \psi_{k_{2}}})+\frac{\partial f(\pi_{t},n_{t},y_{t})}{\partial \pi_{t}}\frac{\partial^{2}\pi_{t}}{\partial \psi_{k_{1}}\partial \psi_{k_{2}}}\right],k_{1}\neq k_{2} \end{array} \end{array}$$

where *k* = 1, 2, 3 and *t* = 1, 2, …, *T*. In addition, from equation 3 we get,

(A3) $$\begin{array}{l} \{\begin{array}{c} \frac{\partial f(\pi_{t},n_{t},y_{t})}{\partial \pi_{t}}=\frac{y_{t}}{\pi_{t}}-\frac{n_{t}-y_{t}}{1-\pi_{t}}\\ \frac{\partial^{2}f(\pi_{t},n_{t},y_{t})}{\partial \pi_{t}^{2}}=-\frac{y_{t}}{\pi_{t}^{2}}-\frac{n_{t}-y_{t}}{{\left(1-\pi_{t}\right)}^{2}}. \end{array} \end{array}$$

For convenience, let

(A4) $$\begin{array}{l} \Omega =\frac{\pi_{0}-\pi_{t}}{\pi_{0}}\Rightarrow \pi_{t}=\pi_{0}(1-\Omega ), \end{array}$$

from both equations A2 and A3, we finally get, for π_0_

(A5) $$\begin{array}{l} \{\begin{array}{c} \frac{\partial \pi_{t}}{\partial \pi_{0}}=1-e^{-e^{\beta t+\alpha }}=\frac{\pi_{t}}{\pi_{0}}=1-\Omega \\ \frac{\partial^{2}\pi_{t}}{\partial \pi_{0}^{2}}=0 \end{array} \end{array}$$

and for β,

(A6) $$\begin{array}{l} \{\begin{array}{l} \frac{\partial \pi_{t}}{\partial \beta }=\pi_{0}t\cdot e^{\beta t+\alpha }\cdot e^{-e^{\beta t+\alpha }}=t(\pi_{0}-\pi_{t})\cdot \text{log}\frac{\pi_{0}}{\pi_{0}-\pi_{t}}=-t\pi_{0}\Omega \cdot \text{log}\Omega \\ \begin{array}{l} \frac{\partial^{2}\pi_{t}}{\partial \beta^{2}}=\pi_{0}t^{2}\cdot e^{\beta t+\alpha }\cdot e^{-e^{\beta t+\alpha }}\cdot (1-e^{\beta t+\alpha })=t^{2}(\pi_{0}-\pi_{t})\cdot \text{log}\frac{\pi_{0}}{\pi_{0}-\pi_{t}}\cdot (1-\text{log}\frac{\pi_{0}}{\pi_{0}-\pi_{t}})\\ \;=-t^{2}\pi_{0}\Omega \cdot \text{log}\Omega \cdot (1+\text{log}\Omega ) \end{array} \end{array} \end{array}$$

and for α,

(A7) $$\begin{array}{l} \{\begin{array}{l} \frac{\partial \pi_{t}}{\partial \alpha }=\pi_{0}\;\cdot \;e^{\beta t+\alpha }\;\cdot \;e^{-e^{\beta t+\alpha }}=(\pi_{0}-\pi_{t})\;\cdot \text{ log}\frac{\pi_{0}}{\pi_{0}-\pi_{t}}=-\pi_{0}\Omega \;\cdot \text{ log}\Omega \\ \begin{array}{l} \frac{\partial^{2}\pi_{t}}{\partial \alpha^{2}}=\pi_{0}\;\cdot \;e^{\beta t+\alpha }\;\cdot \;e^{-e^{\beta t+\alpha }}\cdot (1-e^{\beta t+\alpha })=(\pi_{0}-\pi_{t})\;\cdot \text{ log}\frac{\pi_{0}}{\pi_{0}-\pi_{t}}\;\cdot \;(1-\text{log}\frac{\pi_{0}}{\pi_{0}-\pi_{t}})\\ \;=-\pi_{0}\Omega \;\cdot \text{ log}\Omega \;\cdot \;(1+\text{log}\Omega ) \end{array} \end{array} \end{array}$$

and the derivatives for cross terms are,

(A8) $$\begin{array}{l} \{\begin{array}{l} \frac{\partial^{2}\pi_{t}}{\partial \pi_{0}\partial \beta }=t\cdot e^{\beta t+\alpha }\cdot e^{-e^{\beta t+\alpha }}=\frac{t(\pi_{0}-\pi_{t})}{\pi_{0}}\cdot \text{log}\frac{\pi_{0}}{\pi_{0}-\pi_{t}}=-t\Omega \cdot \text{log}\Omega \\ \frac{\partial^{2}\pi_{t}}{\partial \pi_{0}\partial \alpha }=e^{\beta t+\alpha }\cdot e^{-e^{\beta t+\alpha }}=\frac{\pi_{0}-\pi_{t}}{\pi_{0}}\cdot \text{log}\frac{\pi_{0}}{\pi_{0}-\pi_{t}}=-\Omega \cdot \text{log}\Omega \\ \begin{array}{l} \frac{\partial^{2}\pi_{t}}{\partial \beta \partial \alpha }=\pi_{0}t\cdot e^{\beta t+\alpha }\cdot e^{-e^{\beta t+\alpha }}\cdot (1-e^{\beta t+\alpha })=t(\pi_{0}-\pi_{t})\cdot \text{log}\frac{\pi_{0}}{\pi_{0}-\pi_{t}}\cdot (1-\text{log}\frac{\pi_{0}}{\pi_{0}-\pi_{t}})\\ \;=-t\pi_{0}\Omega \cdot \text{log}\Omega \cdot (1+\text{log}\Omega ) \end{array} \end{array} \end{array}$$

Therefore, the Fisher information matrix is,

(A9) $$\begin{array}{l} {}[I_{j}{\;}_{k}(\pi_{0},\beta ,\alpha )]=-E\left[\frac{\partial^{2}l(\pi_{0},\beta ,\alpha )}{\partial \psi_{j}\partial \psi_{k}}\right]. \end{array}$$

Take equation A2 into equation A9, and we have,

(A10) $$\begin{array}{l} {}[I_{j}{\;}_{k}(\pi_{0},\beta ,\alpha )]=-E\left[\sum_{t=1}^{T}\left[\frac{\partial^{2}f(\pi_{t},n_{t},y_{t})}{\partial \pi_{t}^{2}}\left(\frac{\partial \pi_{t}}{\partial \psi_{k_{1}}}\frac{\partial \pi_{t}}{\partial \psi_{k_{2}}}\right)+\frac{\partial f(\pi_{t},n_{t},y_{t})}{\partial \pi_{t}}\frac{\partial^{2}\pi_{t}}{\partial \psi_{k_{1}}\partial \psi_{k_{2}}}\right]\right]\\ \;=-\sum_{t=1}^{T}\left[E\left[\frac{\partial^{2}f(\pi_{t},n_{t},y_{t})}{\partial \pi_{t}^{2}}\right]\left(\frac{\partial \pi_{t}}{\partial \psi_{k_{1}}}\frac{\partial \pi_{t}}{\partial \psi_{k_{2}}}\right)+E\left[\frac{\partial f(\pi_{t},n_{t},y_{t})}{\partial \pi_{t}}\right]\frac{\partial^{2}\pi_{t}}{\partial \psi_{k_{1}}\partial \psi_{k_{2}}}\right]. \end{array}$$

Since

(A11) $$\begin{array}{l} \{\begin{array}{l} \begin{array}{l} E\left[\frac{\partial f(\pi_{t},n_{t},y_{t})}{\partial \pi_{t}}\right]=E\left[\frac{y_{t}}{\pi_{t}}-\frac{n_{t}-y_{t}}{1-\pi_{t}}\right]\\ \;=\frac{n_{t}\cdot \pi_{t}}{\pi_{t}}-\frac{n_{t}-n_{t}\cdot \pi_{t}}{1-\pi_{t}}=0 \end{array}\\ \begin{array}{l} E\left[\frac{\partial^{2}f(\pi_{t},n_{t},y_{t})}{\partial \pi_{t}^{2}}\right]=E\left[-\frac{y_{t}}{\pi_{t}^{2}}-\frac{n_{t}-y_{t}}{{\left(1-\pi_{t}\right)}^{2}}\right]\\ \;=-\frac{n_{t}\cdot \pi_{t}}{\pi_{t}^{2}}-\frac{n_{t}-n_{t}\cdot \pi_{t}}{{\left(1-\pi_{t}\right)}^{2}}=-\frac{n_{t}}{\pi_{t}(1-\pi_{t})}, \end{array} \end{array} \end{array}$$

the Fisher information matrix could be simplified to

(A12) $$\begin{array}{l} {}[I_{j}{\;}_{k}(\pi_{0},\beta ,\alpha )]=\sum_{t=1}^{T}\frac{n_{t}}{\pi_{t}(1-\pi_{t})}\cdot \left(\frac{\partial \pi_{t}}{\partial \psi_{k_{1}}}\frac{\partial \pi_{t}}{\partial \psi_{k_{2}}}\right). \end{array}$$
